# Translumbar type II endoleak embolization with a new liquid iodinated polyvinyl alcohol polymer: Case series and review of current literature

**DOI:** 10.3389/fradi.2023.1145164

**Published:** 2023-05-09

**Authors:** Giovanni Leati, Francesco Di Bartolomeo, Gabriele Maffi, Luca Boccalon, Domenico Diaco, Edoardo Segalini, Angelo Spinazzola

**Affiliations:** ^1^ Unit of Interventional Radiology, Ospedale Maggiore di Crema, Crema, Italy; ^2^ Department of Vascular Surgery, Ospedale Maggiore di Crema, Crema, Italy; ^3^ Department of General Surgery, Ospedale Maggiore di Crema, Crema, Italy

**Keywords:** type II endoleak, translumbar embolization, liquid PVA, direct sac puncture, feeders embolization, liquid embolic agents, abdominal aort aneurysm

## Abstract

**Purpose:**

To describe our experience with the use of a novel iodized Polyvinyl Alcohol Polymer liquid agent (Easyx) in type II endoleak treatment with translumbar approach.

**Methods:**

Our case series is a retrospective review of patients with type II endoleak (T2E) treated with Easyx from December 2017 to December 2020. Indication for treatment was a persistent T2E with an increasing aneurysm sac ≥5 mm on computed tomography angiography (CTA) over a 6-month interval. Technical success was defined as the embolization of the endoleak nidus with reduction or elimination of the T2E on sequent CTA evaluation. Clinical success was defined as an unchanged or decreased aneurysm sac on follow-up CTA. Secondary endpoints included the presence of artifacts in the postprocedural cross-sectional tomographic imaging and post and intraprocedural complications.

**Results:**

Ten patients were included in our retrospective analysis. All T2E were successfully embolized. Clinical success was achieved in 9 out of 10 patients (90%). The mean follow-up was 14 3–20 months. No beam hardening artifact was observed in follow-up CT providing unaltered imaging.

**Conclusion:**

Easyx is a novel liquid embolic agent with lava-like characteristics and unaltered visibility on subsequent CT examinations. In our initial experience, Easyx showed to have all the efficacy requisites to be an embolization agent for type II EL management. Its efficacy, however, should be evaluated in more extensive studies and eventually compared with other agents.

## 1. Introduction

The endovascular approach has become the mainstay therapy for treating abdominal aortic aneurysms. Compared to the surgical approach, the short-term advantage in terms of mortality is later jeopardized by complications such as endoleaks, which require a tighter follow-up. T2E is defined as retrograde filling of the aneurysm sac through patent aortic branch vessels, mostly lumbar or inferior mesenteric arteries. T2E is the most frequent complication following endovascular aneurysm repair (EVAR) (1) and the first cause (up to 16% of patients) (2) of secondary interventions. Reported rates of T2E broadly range from 7% to 44% (1–3). While more than half of T2E seal spontaneously, there is a small risk of sac rupture when endoleaks persist or occur lately (4–6). T2E can be corrected with different endovascular approaches with transarterial (through the Riolan arcade or iliolumbar artery) and direct sac puncture (transabdominal, translumbar, or transcaval) accounting for most patients (7–9). Surgical correction is nowadays reserved for a small subset of patients with failed endovascular approach. Embolic agents include coils and liquid embolic agents alone or in combination. Nowadays, coils seem not to be the best material in long terms follow-up (10), and there's a shift to liquid embolic, especially NBC (*N*-butyl cyanoacrylate) and EVOH-based liquids like Onyx (Onyx® LES, Covidien, Plymouth, MN, USA) that are the most used. Although literature lacks RCTs (randomized controlled trials) and there is no consensus about the best embolic agent, few comparative studies reported a reduction of reintervention with NBC (11) and Onyx (10) rather than coils alone. Onyx has been used in one of the largest studies on patients treated *via* direct sac puncture (12). Moreover, a more recent study demonstrated no improvement in treating T2E with Onyx in case of recurrence after first embolization (13).

Our series aims to describe our preliminary experience and to review the existing literature about translumbar embolization of persistent T2E with a novel iodized liquid Polyvinyl Alcohol (PVA) polymer agent (EASYX, Qmedics AG, Flurlingen, Switzerland). This new embolic agent (with CE approval for peripheral use) consists of a PVA Polymer in liquid form which forms a solid cast when the solvent (Dimethyl sulfoxide—DMSO) dissipates in contact with the bloodstream. This liquid agent has no adhesive capacity, and it is covalently bonded with iodine, which provides its intrinsic opacity.

## 2. Materials and methods

### 2.1. Patients

In our case series, we retrospectively analyzed patients who underwent translumbar T2E embolization with Easyx alone or in combination with coils between December 2017 and January 2020. Patient data was obtained through the review of imaging reports and medical records. According to institutional statutes, no ethical clearance for such retrospective analysis is required.

Procedure indication, patient selection, and the easier approach (translumbar or transarterial) were discussed by interventional radiologists and vascular surgeons during a multidisciplinary team meeting. All patients had a persistent T2E with an increase of the aneurysm sac ≥5 mm on at least one out of 2 axial diameters measured on CTA in a minimum interval of 6 months. All CT scans were performed on the same machine (Toshiba Aquilion 64 slice, Tokyo, Japan) and included a non-enhanced, arterial, and venous phase. Images were analyzed with the integrated PACS software (Philips intellispace PACS enterprise 4.4, Philips, Netherlands) by a radiologist with at least 5 years experience in vascular imaging.

Technical success was defined as the embolization of the endoleak nidus with reduction or elimination of the T2E on sequent CTA evaluation. Clinical success was defined as an unchanged or decreased aneurysm sac on a follow-up CTA made during the follow-up, with the first one made after 6 months.

### 2.2. Procedure

All embolization procedures were performed in the same angio suite (Siemens Artis, Siemens Healthineers, Erlangen, Germany) under local anesthesia by one interventional radiologist and one vascular surgeon (rotating among 3 interventional radiologists and 4 vascular surgeons).

Sedation was reserved for especially anxious patients in order to reduce movements, and analgesic drugs were administered to control back pain in some patients.

**Figure 1:**
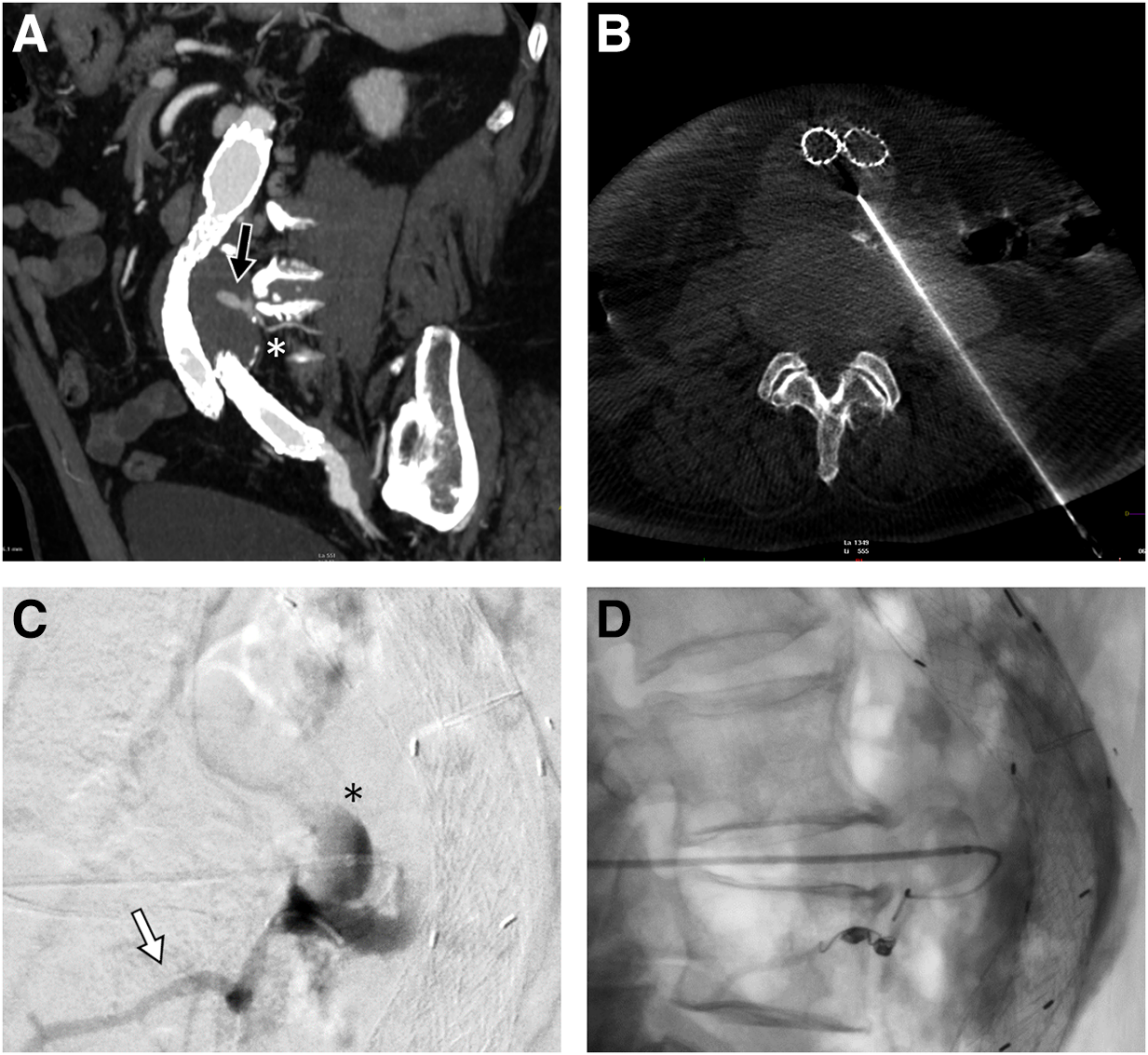
Sagittal MIP of the pre-procedural CTA showing the posterior endoleak (black arrow) along with the lumbar feeder (white asterisk) (**A**). Cone beam CT confirming the 21G needle tip positioning inside the aneurysmatic sac (**B**). In lateral projection, the injection from the microcatheter shows the endoleak nidus (black asterisk) and the lumbar feeder (white arrow) (**C**), which is embolized with detachable microcoils (**D**).

**Figure 2:**
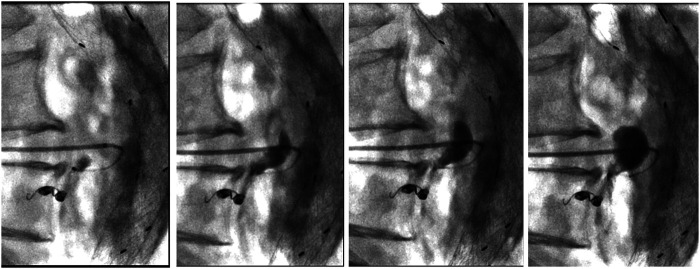
Progressive filling of the endoleak with easyx (from left to right). The tip of the microcatheter is located near the embolized inflow vessel and inside the embolic cast, but it is effortlessly removed 10 min later.

Written consent was obtained from the patient before each procedure. We standardized the procedure in order to have stable access into the sac with an introducer sheath: in this way, we could both navigate the feeders with different catheter shapes and safely inject the DMSO through a distally placed compatible microcatheter. We used a tapered 4 French introducer system with radiopaque marker and a coaxial system including the coaxial dilator with metallic stiffening cannula (Accustick, Boston Scientific, Marlborough, USA) which facilitates over-the-wire translumbar placement. The aneurysm sac was punctured on the left side at the level of the T2E nidus with a 21 Gauge, 15 cm long needle with stylet under fluoroscopic and cone beam CT (CBCT) guidance with the patient lying in a prone position (Figure 1B). A 0.18 stainless steel guidewire was pushed into the aneurysmatic sac, and the introducer system was advanced on it. Contrast media was injected through a 4 French catheter (Bernstein curve) advanced into the aneurysmatic sac in order to evaluate endoleak anatomy and its feeders (Figure 1C). Selective catheterization of the feeders was attempted through a DMSO compatible microcatheter (Progreat 2, 4 Fr 130 cm length, Terumo, Tokyo, Japan) and, when successful, they were embolized with detachable micro-coils (Concerto coils Medtronic, Minneapolis, USA) with a diameter ranging from 3 to 4 mm (Figure 1D). Once the lumbar arteries were embolized, or in case of unsuccessful catheterization, the microcatheter dead space was gently flushed with DMSO and then endoleak nidus was filled with Easyx under fluoroscopic control (Figure 2). No coils were deployed inside the aneurysm sac.

At the end of the procedure, the coaxial system was removed, and gentle compression was applied at the entry point for 5 min. Post-procedure CBCT was not performed systematically. After embolization, patients were admitted to the vascular surgery department and monitored for at least 24 h before discharge. A follow-up CTA was scheduled at 6 months for all the patients.

### 2.3. Statistics

Patient and procedure data have been retrospectively collected in a database. Descriptive statistical analysis of significant clinical data was performed on the dataset using Microsoft Excel 2011 (Microsoft Corporation, Redmond, WA, USA). Most representative data are reported with mean and range.

## 3. Results

Between December 2017 and December 2020, we performed 13 embolization procedures for persistent T2E. Three patients treated with transarterial approach were excluded (2 through the inferior mesenteric artery and one through the iliolumbar artery) in order to focus our study only to direct percutaneous sac puncture. We eventually selected 10 consecutive patients (9 men; mean age: 76, range: 64–84) reported in Table 1, who underwent T2E through the translumbar approach. Two of these patients had already been treated for T2E with iliolumbar embolization before December 2017, respectively 24 and 36 months before translumbar embolization using coils and thrombin. The average time from EVAR procedure to endoleak treatment was 23 months (range: 6–47 months). Lumbar feeders were successfully engaged and occluded in 5/10 patients (50%, total of 8 lumbar feeders). A total of 19 vials (each containing 1.3 ml) of Easyx were used in 10 patients (min-max 1–4, mean 1, 9). In 4 patients 1 vial was used, in 4 patients 2 vials, in 1 patient 3 vials, and in 1 patient 4 vials.

**Table 1:** Patients data collection.

Sex	Age	Aneurysm size	sac expansion (mm)	n° vials (corresponding volume, ml)	Number of lumbar arteries embolized	Clinical success	Technical success	Fu months
m	82	77 × 56	6	1 (1,3)	0	Yes	Yes	12
m	80	52 × 49	7	3 (3,9)	0	Yes	Yes	30
m	67	60 × 47	14	4 (5,2)	2	Yes	Yes	26
m	77	125 × 113	8	1 (1,3)	0	No	Yes	3
m	76	47 × 52	6	2 (2,6)	2	Yes	Yes	16
m	84	43 × 46	8	1 (1,3)	0	Yes	Yes	14
m	64	71 × 67	7	1 (1,3)	2	Yes	Yes	6
m	80	64 × 62	5	2 (2,6)	2	Yes	Yes	8
m	75	82 × 85	7	2 (2,6)	2	Yes	Yes	12
f	68	87 × 76	6	2 (2,6)	0	Yes	Yes	12

The mean total procedure time was 93 min (min-max 41–130), and fluoroscopic duration was 28 min (min-max 15–54). The mean Dose Area Product was 50.892 µGym^2^ (min-max 20,316–77,473). The mean follow-up was 14 months (range, 3–30 months).Technical success was achieved in all 10 cases (100%). Clinical success was achieved in 9/10 patients (90%): one patient had a reduced but persistent T2E and eventually underwent open conversion with endograft resection 3 months later, after a CTA re-evaluation. In this case, the indication for open conversion was persistent back pain without neurological or musculoskeletal cause, which was therefore attributed to the still pressurized aneurysm sac (10.8 cm). After surgical correction, back pain resolved, and the patient was still alive at follow-up. In one patient we fluoroscopically observed a small amount of embolic material adjacent to the aneurysm sac. This was later drained into the vena cava and subsequently caused a small subsegmental lung embolism. The embolism was asymptomatic and required no additional treatment.

**Figure 3:**
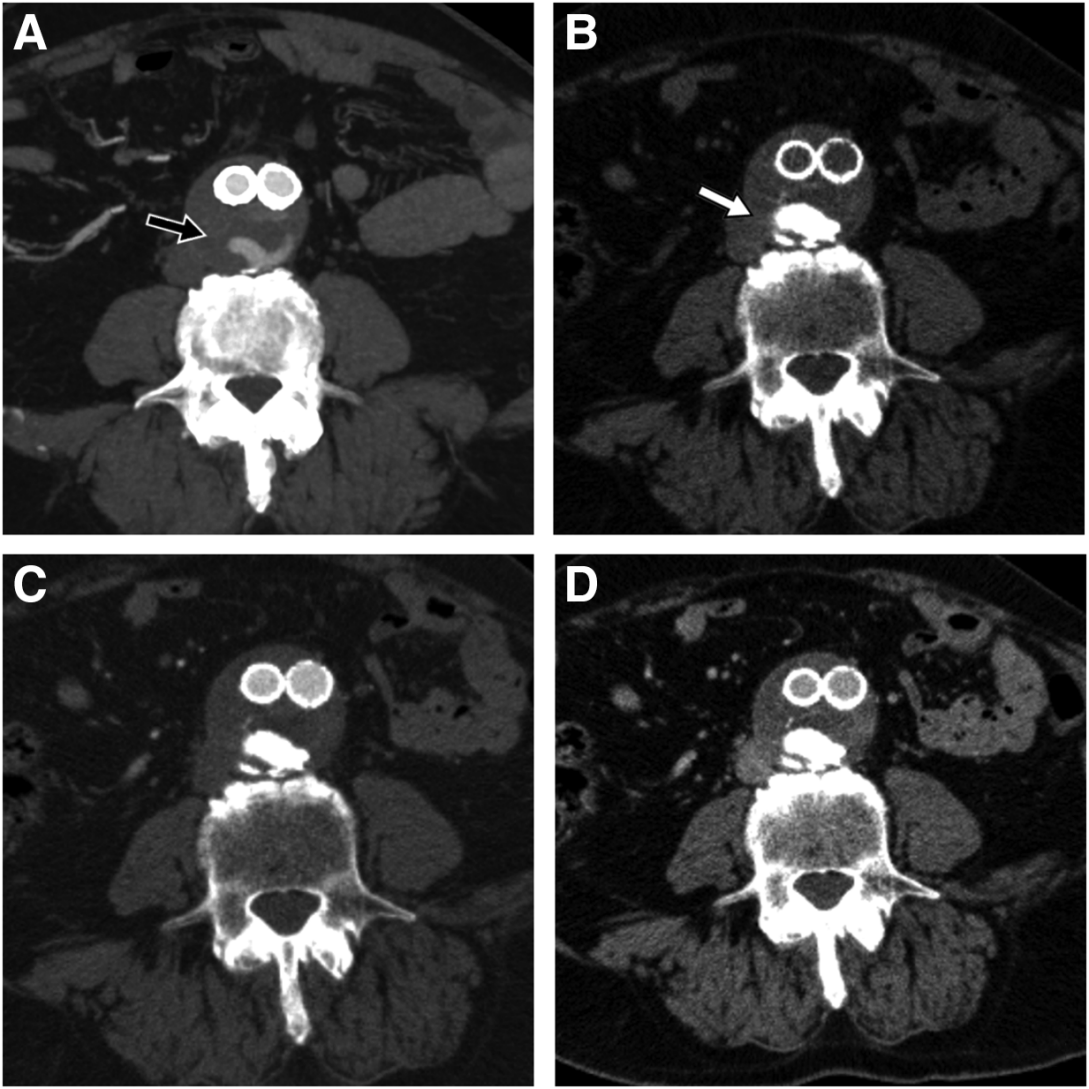
Pre-procedural CTA showing part of the endoleak during arterial phase (black arrow) (**A**), 6-month follow-up CTA on the same anatomic level showing easyx cast in unenhanced (white arrow) (**B**), arterial (**C**), and venous phase (**D**) with complete disappearance of the T2E.

Two patients were lost to follow-up at 6 and 12 months without evidence of T2E at the control CTA. Three patients (30%) experienced back pain related to the prolonged prone position, which resolved at the end of the procedure. No beam hardening artifacts were observed in the follow-up CTAs (Figure 3).

## 4. Discussion

T2E treatment is still a debated argument in the scientific community with a variety of management strategies. While there is a growing number of studies suggesting that conservative management could be an option even for T2E with growing sac with no difference in terms of mortality (14, 15), a persistence of T2E is associated with an increased rate of interventions, surgical conversion, and adverse late outcomes (5, 16). In major guidelines (4, 6), a sac enlargement of more than 5 mm over a 6-month interval is currently an accepted indication for embolization. This heterogeneity in T2E management is also observable in the technical approach to the procedure. Different treatment strategies include: transarterial route through the Riolan arcade (when the inferior mesenteric artery is involved) or through iliolumbar arteries and direct sac access that can be achieved *via* translumbar, transcaval (17), perigraft (18) or transabdominal puncture (7). The choice of embolic material ranges from coils to liquid agents like cyanoacrylate glue, thrombin, non-adhesive embolic agents, or their combination.

Material choice is mainly influenced by operator confidence in it and by the fact that none of these agents managed to achieve a significant clinical and technical benefit over the others. Reported experience with liquid embolic agent use in T2E is heterogeneous in terms of technique and combination of materials used and includes almost exclusively ethylene vinyl alcohol copolymer (EVOH) and in particular Onyx (Onyx® LES, Covidien, Plymouth, MN, USA). The other EVOH agent (Squid, Balt, Montmorency, France) use is only reported among different peripheral use (19) (4 out of 30 patients) and without reported follow-up. First Easyx use in humans was recently reported by Sapoval et al. (20) and includes results at 6 months follow-up for 7 endoleak procedures among 50 patients. Literature about Precipitating hydrophobic injectable liquid (PHIL; Micro-Vention, Tustin, California) use in T2E is limited to small case series with three patients (21). No randomized controlled trial comparing different liquid agents is available at the moment of submission. In terms of efficacy, the clinical (90%) and technical (100%) success rates we observed are comparable and, in some cases, slightly superior to the other series reporting T2E embolization with liquid embolic agents. The slight superiority observed over other series is likely biased and mainly related to the short follow-up and to the small cohort of patients of our series and, for these reasons, must be validated and eventually re-compared with longer follow-up and a more significant cohort. The data relevant to the comparison of these series are summarized in Table 2. In 6 out of 7 case series, the liquid embolic agent has been used in combination with coils. In 4 out of 7 series, the subsequent follow-up CTAs are hindered by strike artifacts while in the other two, this aspect is not addressed. The embolization technique used is mixed (transarterial or direct puncture) in 4 studies, exclusively transarterial in 2, and exclusively translumbar in one study.

**Table 2:** 

Author	Patients	Material	Technique	Mean follow up (months)	Technical success	Clinical success	Complications	Artifacts
Khaja et al	18	Onyx + coils + glue + plug	Direct sac puncture + transarterial	32	88%	88%^1^	3 (transient L2 nerve paralysis, intraperitoneal onyx leak, psoas hematoma)	Strike artifacts
Wojtaszek et al	22	Onyx + coils (feeders)	Transarterial	17	81%	80%	1 (inflammatory thickening of the aortic wall seen on follow-up images)	Strike artifacts
Marcelin et al	28	Onyx + coils (feeders)	Direct sac puncture + transarterial	20	72/14%^2^	primary 75% secondary 96%		Not reported
Muller-Wille et al	11	Onyx + coils (feeders)	Transarterial	26	55%^3^	73%		Strike artifacts
Yu et al	29	Onyx + coils + glue	Direct sac puncture + transarterial	20/24	85%	62/61%^4^		Strike artifacts
Ierardi et al	12	Onyx + glue + thrombin	Direct sac puncture + transarterial	27	100%	91%		Not reported
Fanelli et al	50	Onyx + Coils	Direct sac puncture	12	100%	100%^5^	None	Not reported^6^

^1^ With 8 subsequent reinterventions.

^2^ Respectively for transarterial or direct sac puncture group.

^3^ Technical defined as complete nidus embolization.

^4^ Patients divided in nidus embolization only group and nidus + feeders embolization group.

^5^ There is no definition of clinical success in this article, but if we considered clinical success as sac shrinkage or stability, it is 100%.

^6^ Follow up with CEUS.

At our institution, we use the transarterial or the direct translumbar puncture approach, the easiest route is chosen according to the operator and each case is evaluated individually during a multidisciplinary meeting. Since its introduction into the market in 2017, we have used Easyx in different clinical settings (AVM, bleeding embolization, venous embolization, benign tumors) and as the preferred liquid embolic agent for T2E embolization.

**Figure 4:**
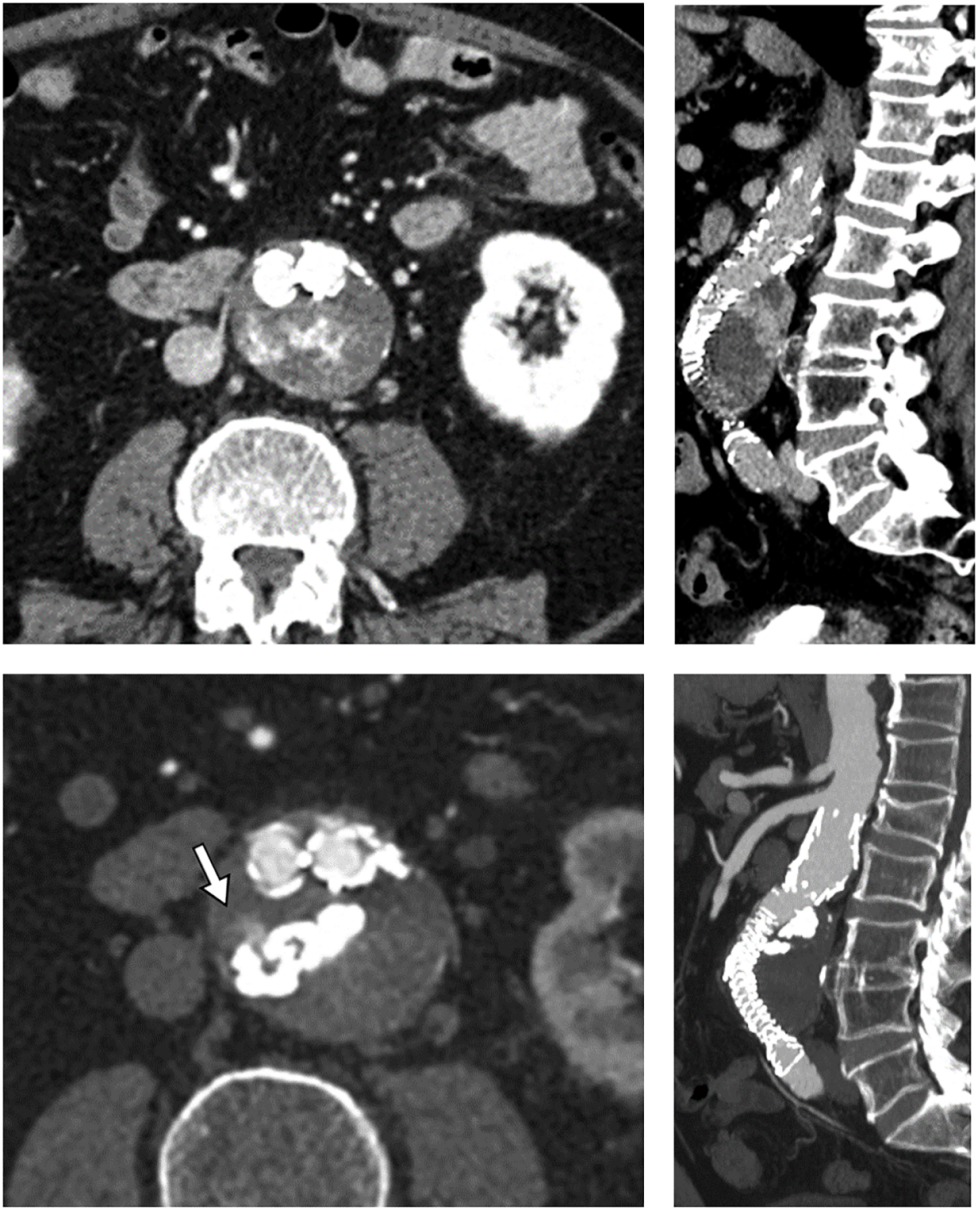
Pre- and post-procedural images (respectively top and bottom, axial and sagittal MPR) showing the easyx cast with the persistence of a small portion of the endoleak adjacent to it (white arrow). This endoleak had no clinical significance for the patient.

The reason is mainly due to a combination of beam hardening artifact absence on follow-up CTA, “lava like” consistency, and the intrinsic non-adhesive nature of the liquid PVA as reported by Kulcsár et al. (22). In their animal study, the density observed on follow-up CT was close to cortical bone one (600–800 HU), without any beam hardening artifact. This feature is confirmed in our T2E series using Easyx and by Sapoval et al. (20). Easyx radiopacity is provided by covalently bonded iodine. The main advantage of iodine density is the absence of beam hardening artifacts on follow-up CT (20, 22) (especially when a large amount of embolic material is present) which preserved the diagnostic quality of the post-procedural CTAs. This latter characteristic can be useful in this subset of EVAR patients who undergo several follow-up CTAs. A precise evaluation can be done in case of persistent endoleak (Figure 4) to evaluate recruitment of new feeders or changed T2E hemodynamic when a reintervention is needed. Another advantage of the covalent bond of the iodine group is that the embolic agent is ready to use without the need for pre-procedural homogenization of the vial (22) and without risk of precipitation of the tantalum suspension inside the syringe or microcatheter (23).

**Figure 5:**
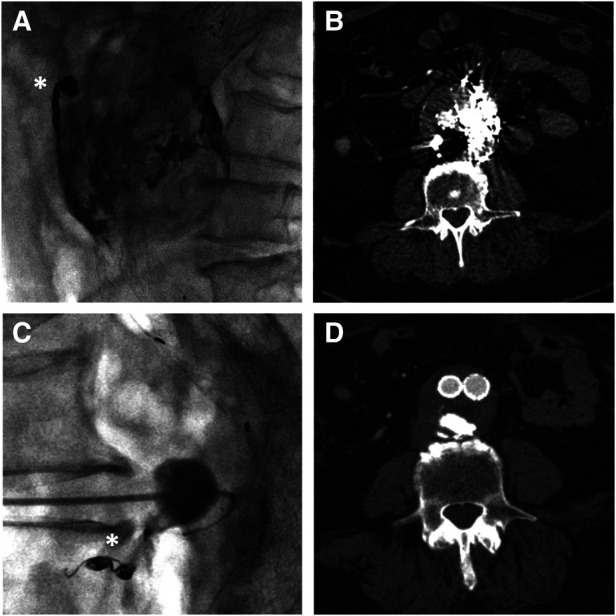
Radiopacity and strike artifacts comparison between onyx (top, figures (**A,B)**, containing tantalum) and easyx (bottom, figures (**C,D)**, containing iodine) on two type II endoleak embolization on fluoroscopy (**A–C**) and CTA follow-up (**B–D**). Coils in fluoroscopic images (**A–C**) are indicated by white asterisks.

**Figure 6:**
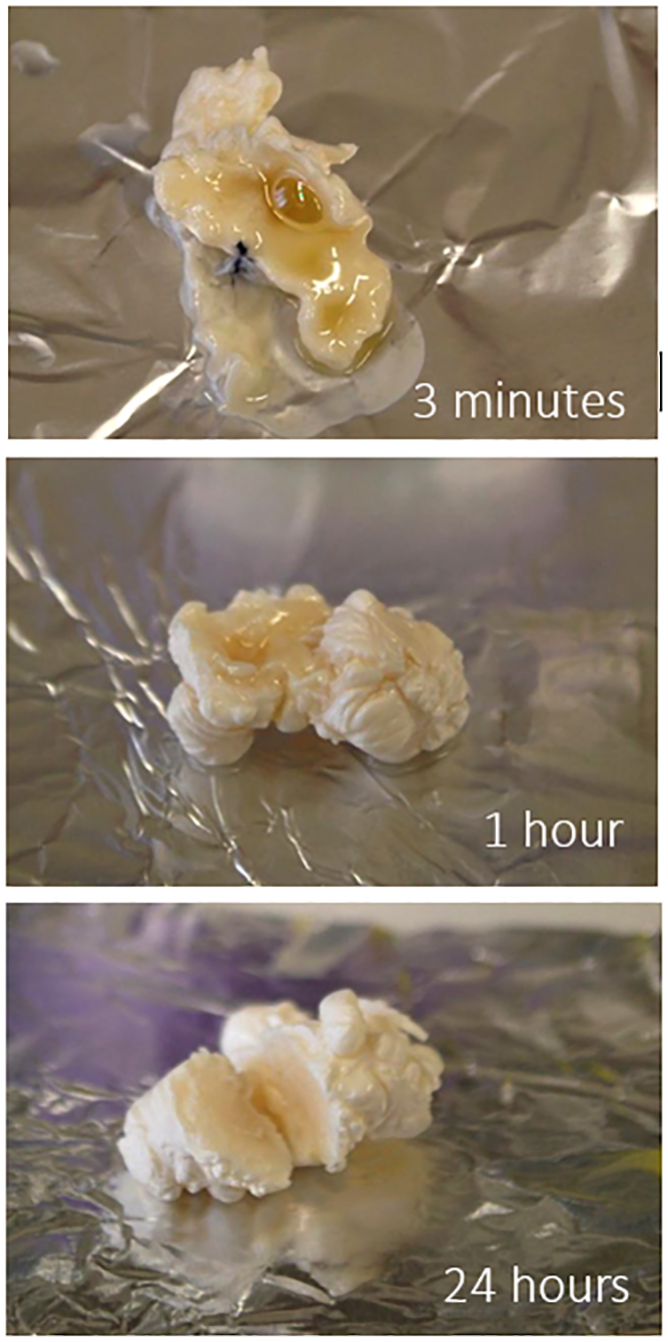
The liquid core of easyx at different time frames during a bench test.

**Figure 7:**
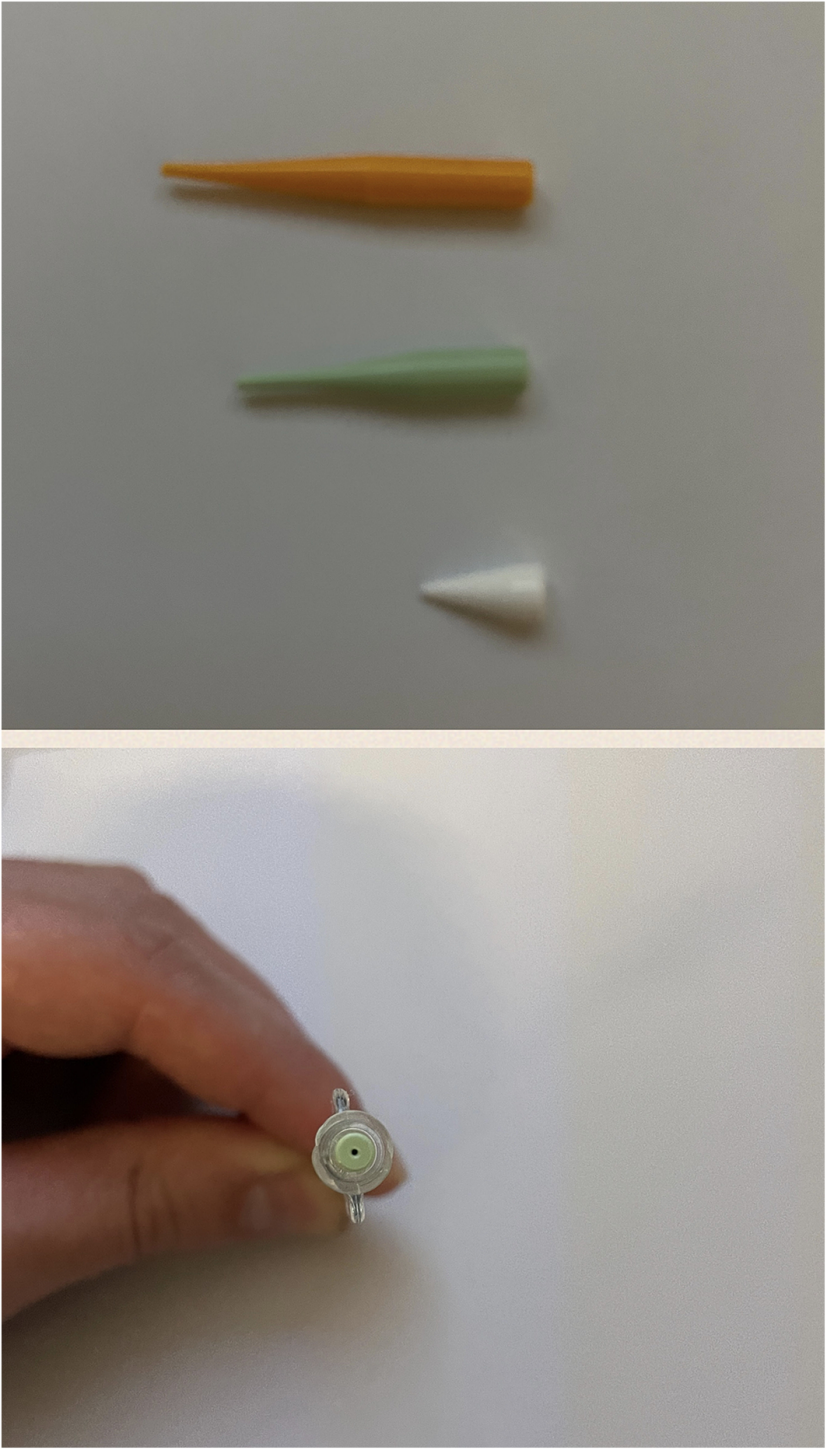
Hub adapters and usage representation.

On the other side, fluoroscopic visibility provided by iodine is slightly inferior to other agents containing tantalum (Figure 5) especially when large portions of the abdomen need to be traversed by x-ray in lateral or oblique projection. This limit can be overcome by adequate imaging equipment and image optimization, like the use of lower Kilovolt kV and proper collimation in order to maximize image contrast, albeit at the expense of image noise (24). The “lava-like” consistency is characterized by an external polymerized surface with a liquid core that is extruded during prolonged injection and eventually fills the empty space in the endoleak nidus. This can be achieved only by non-fragmentable embolic agents with a slow polymerization time and without adhesive properties. This feature is essential when the aim of the procedure is the complete filling of the endoleak “nidus” and not just feeders occlusion, which can eventually lead to EL recurrence through a different route (25). From bench tests, the liquid core of Easyx is observable even after 24 h from the initial polymerization (Figure 6). In terms of safety, among the 58 injections performed, Kulcsár et al (22). observed one distal unnoticed fragmentation of the cast and assumed that the non-target embolization was due to the Easyx-DMSO overdilution in the microcatheter hub. This led to a mixture of too low a viscosity which let the embolic agent migrate too distally. This complication was subsequently avoided by limiting the microcatheter hub dead space with a dedicated hub adapter (Figure 7). In our series, we observed one complication as well due to non-target embolization leading to a small subsegmental lung embolism. Although this complication has been already reported once (26), the underlying mechanism has not been assumed. Based on the follow-up CTA, we think that this complication is due to an unintentional microwire perforation of the aneurysm wall with a subintimal wedged injection of embolic material which was eventually drained into small veins converging in the cava. In addition to the microcatheter displacement, the Easyx-DMSO hub overdilution could have had a role in our complication too. As Kulcsár et al. (22) did, for the subsequent procedures we introduced the use of the dedicated microcatheter hub adapter (included in the product package) (Figure 7) in order to reduce the microcatheter dead space.

Since then, we have not observed any further non-target embolization in any procedure (endoleak, trauma, AVM) with Easyx. Therefore, we think that this complication is not related to the embolic material itself but rather to the operator learning curve and to the microcatheter malpositioning. The material radiopacity was adequate to promptly recognize the non-target embolization and immediately stop the injection.

Finally, we did not observe any microcatheter entrapment even after prolonged injection from the microcatheter submerged into the embolic cast (Figure 2). The absence of microcatheter entrapment, also reported by Kulcsár et al. (22), is enabled by the intrinsic non-adhesive nature of the liquid PVA. The cost of Easyx in our hospital is slightly less expensive than the other product in the same category we have (Onyx).

This study is limited by the small cohort of patients and by its retrospective and observational nature. It is therefore limited by the fact it's monocentric and could be influenced by operator bias. Some of the shortcomings of the study could be overcome by larger and randomized studies comparing this agent with the others present on the market. The limited mean follow-up is mainly due to the recent introduction of this embolization agent in the market.

## 5. Conclusion

Our preliminary experience with this new embolic agent in the T2E procedure has been positive in terms of technical and short-term clinical success. Non-artifacted follow-up CT certainly adds value to the comprehension of T2E evolution after the embolization procedure.

Easyx-DMSO overdiluition in the microcatheter hub must be carefully avoided in order to prevent non-target embolization. Given the small number of patients, the limited follow-up, and the retrospective nature of this work, larger studies will be required in order to better assess the efficacy of this agent in this and other types of embolic procedures.

## Data Availability

The raw data supporting the conclusions of this article will be made available by the authors, without undue reservation.
